# Editorial: Experts' opinion in public health education and promotion

**DOI:** 10.3389/fpubh.2023.1211391

**Published:** 2023-05-23

**Authors:** Christiane Stock

**Affiliations:** ^1^Charité - Universitätsmedizin Berlin, Corporate Member of Freie Universität Berlin and Humboldt-Universität zu Berlin, Institute of Health and Nursing Science, Berlin, Germany; ^2^University of Southern Denmark, Unit for Health Promotion Research, Esbjerg, Denmark

**Keywords:** health promotion, Ottawa Charter, health education, population health, public health system

In this Research Topic the Public Health Education and Promotion section of Frontiers in Public Health gathers opinions and perspectives on innovative research, education, and practice that will advance the scientific basis of knowledge help inform the public, professionals, and societies to improve the health of the population. The Frontiers Section Public Health Education and Promotion emphasizes protecting and promoting population health by collating contributions to health education and promotion from researchers, educators, decision-makers, and practitioners. In doing so, it is deeply rooted in the principles of the Ottawa Charter for Health Promotion. The Ottawa Charter for Health Promotion released in 1986 by the World Health Organization outlines a holistic approach to health promotion (1) and identifies five key strategies for promoting health and preventing disease: building healthy public policy, creating supportive environments, strengthening community action, developing personal skills, and reorienting health care services toward prevention and health promotion. The Ottawa Charter has had a significant impact on public health policy and practice worldwide (2). Although many social and economic changes have occurred and globalization as well as digitization have had strong impacts on systems, governance structures, economies, the Ottawa Charter for Health Promotion is still very relevant today (3). In fact, many countries continue to use the Charter as a framework for developing public health policies and programs, including initiatives to address non-communicable diseases, mental health, and social determinants of health (4). In addition, the Charter's emphasis on equity and empowerment of individuals and communities is particularly relevant in the context of the COVID-19 pandemic, which has highlighted existing health disparities and the importance of community participation in public health responses (5). The opinion or perspective articles in this Research Topic address different action areas of the Ottawa Charter.

The opinion article of Atalay et al., addresses the prevention paradox resulting from the COVID-19 pandemic, where public health officials implemented quarantine as a protective measure. While quarantine protected vulnerable populations groups from infection it at the same time left women and children to fell victim to increased violence (6). Therewith, the COVID-19 pandemic has also created a pandemic of violence that dramatically increased the number of women abused by their intimate partners and the frequency of violent encounters (7). In order to effectively address this problem in future pandemics or natural disasters Atalay at al. suggest to develop solutions on all levels with formal and informal community-based support networks. But they also recommend to build healthy public policy and to provide financial support to women and to offer housing to women with financial insecurities to combat violence against women in pandemic or disaster situations.

The perspective article of Dadaczynski et al. focusses of the Ottawa Charter's action area of developing personal skills. The authors argue that in pandemic situations games offer great potential for hard to reach population groups to provide up-to date information on various aspects of the pandemic and to communicate recommendations. The article reflects the opportunities and challenges of games for health especially during the pandemic but also with regard to future health crises. As recommendations, the authors call for greater cooperation with commercial game makers to distribute health-related messages via entertainment games and make suggestions how to improve games for health for more effective implementation and use.

While addressing the university as a setting for health promotion, the opinion article of Dietz and Schäfer contributes to the important topic of creating supportive environments. The effective implementation of a settings-based approach to health promotion needs a supportive and enabling political environment and framework. In this context, the article critically discusses the German prevention act to strengthen health promotion and prevention and outlines some recommendations to support German universities on their way to becoming health-promoting institutions.

With respect to the action area of the Ottawa Charter to reorienting health care, the opinion article of Gutiérrez-Barreto and Gutiérrez reflects on the need for a specific healthcare model that suits older population care. The article discusses the Integrated Care for Older People (ICOPE) developed by the World Health Organization (WHO) that uses available evidence to provide countries with micro, meso, and macro implementation actions to guide the services and systems (8). As community-based approach, the ICOPE program aims to strengthen health services by building person-centered long-term care systems with a coordinated model of care. The authors argue that the ICOPE approach requires an in-country assessment of program theory and design to strengthen and guide the local implementation.

To further advance the scientific basis of knowledge helping to inform the public, professionals, and societies to improve the health of the population, further research is needed and will be collected in the Frontiers Section Public Health Education and Promotion. Firstly, there is a need for research that identifies effective interventions for promoting health and preventing disease. This may include evaluating the effectiveness of community-based health promotion programs or examining the impact of policies that address social determinants of health. Secondly, research is needed on the social and environmental factors that influence health outcomes. This includes understanding how factors such as income, education, employment, housing, and racism affect health and wellbeing, and how to address these factors to improve health outcomes. Thirdly, there is a need for research on the cultural, social, and economic contexts in which health promotion strategies are implemented. This can include understanding the perspectives and needs of communities, and identifying ways to engage and empower them in health promotion efforts. Overall, research that is conducted within a multidisciplinary framework, involves community engagement, and addresses the social determinants of health is critical to achieving the aims of the Frontiers Section Public Health Education and Promotion.

## Author contributions

CS conceptualized the editorial article and wrote the manuscript.
